# Supplementary material for: Drought-Stress-Related Reprogramming of Gene Expression in Barley Involves Differential Histone Modifications at ABA-Related Genes
Source: Int J Mol Sci. 2023 Jul 27;24(15):12065. doi: 10.3390/ijms241512065 (PMC10418636; doi:10.3390/ijms241512065)
Supplement: Supplementary file 1 [file ijms-24-12065-s001.zip › ijms-2507846-supplementary.pdf]

Supplemental figures

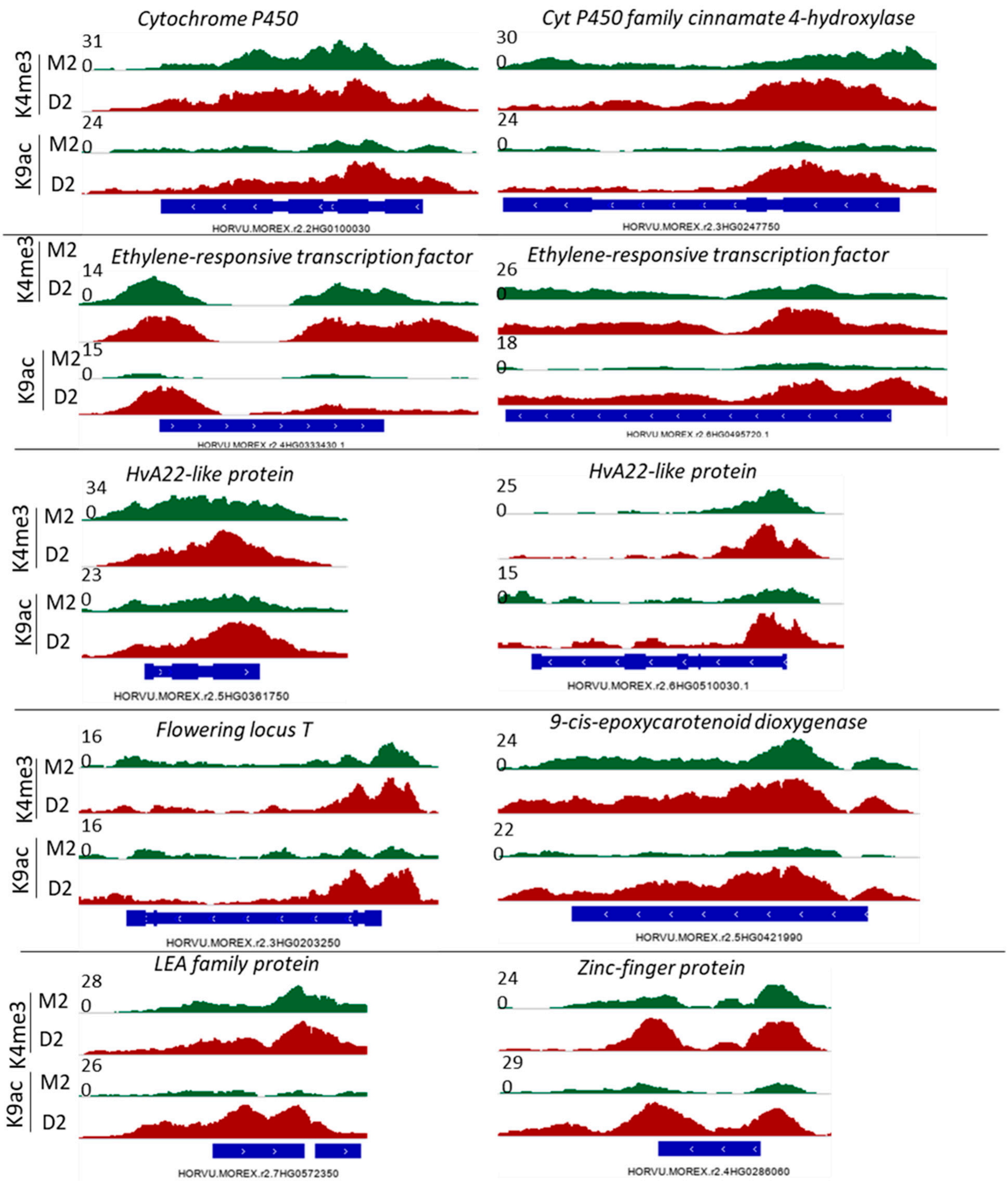

Figure S1. Signal tracks of histone enrichments for selected genes showing an increase in H3K9ac in D2 belonging to the GO term 'response to abiotic stimulus'. First two tracks belonging to H3K4me3 and the two bottom tracks to H3K9ac. Green color presents the M2 sample and red the D2 samples.
